# Implementing and Evaluating Comprehensive Evidence-Based Approaches to Prevent Youth Violence: Partnering to Create Communities Where Youth Are Safe From Violence

**DOI:** 10.1007/s10935-016-0422-y

**Published:** 2016-03-26

**Authors:** Jennifer L. Matjasko, Greta M. Massetti, Sarah Bacon

**Affiliations:** Centers for Disease Control and Prevention, Atlanta, GA USA; Division of Cancer Prevention and Control, Centers for Disease Control and Prevention, Atlanta, GA USA; National Center for Injury Prevention and Control, Centers for Disease Control and Prevention, Atlanta, GA USA; Chapin Hall at the University of Chicago, 1313 East 60th Street, Chicago, IL 60637 USA

**Keywords:** Youth violence prevention, Research-community partnerships, Evidence-based programs, Implementation science, Evaluation of comprehensive strategies

## Abstract

Violence, including its occurrence among youth, results in considerable physical, emotional, social, and economic consequences in the U.S. Youth violence prevention work at the Division of Violence Prevention (DVP) at the Centers for Disease Control and Prevention (CDC) emphasizes preventing youth violence-related behaviors, injuries, and deaths by collaborating with academic and community partners and stakeholders. Since 2000, DVP has funded three rounds of CDC’s National Centers of Excellence in Youth Violence Prevention (YVPCs) in 5-year cycles, with the goal of supporting university-community partnerships so that the best science can be utilized in order to prevent youth violence. The current YVPCs focus on: (a) partnering with communities to identify community needs; (b) selecting and implementing the best comprehensive evidence-based programs to meet those needs; and (c) rigorously evaluating whether those efforts have a community-level impact on youth violence rates. The introduction to this special issue on the current YVPCs provides a brief historical overview on the YVPC Program; outlines the YVPCs’ accomplishments to date; and describes the current YVPCs, their community partners, and their activities. The introduction concludes with an overview of the special issue.

## Introduction

Over the past 15 years, the U.S. has witnessed a general decline in overall rates of youth homicide (David-Ferdon, Dahlberg, & Kegler, 2013). Nonetheless, youth violence rates remain high in this country, with homicide being the third leading cause of death among persons aged 10–24 years (Centers for Disease Control and Prevention [CDC], 2011). While the negative consequences of youth violence are experienced most directly by individuals and families, communities and society also experience the negative effects. Youth violence can affect communities by substantially increasing the cost of health care, reducing productivity, and diminishing property values (Mercy, Butchart, Farrington, & Cerda, 2002). In 2005, it was estimated that the medical care and lost productivity costs associated with youth violence were roughly $17.5 billion (WISQARS, 2010).

Given the public health importance of preventing youth violence, it fits with the overall mission of CDC, which seeks to put science into action in order to protect America from health, safety, and security threats. Broadly, the Division of Violence Prevention (DVP) at CDC is committed to the primary prevention of violence. DVP’s work addresses violence at all stages of the public health model: monitoring violence-related injuries; conducting research on risk and protective factors for violence; developing and evaluating the effectiveness of violence prevention programs and strategies; conducting research on and promoting the widespread adoption and dissemination of evidence-based prevention programs and strategies; and helping state and local partners plan, implement, and evaluate prevention programs and strategies.

Despite DVP’s many state and local partnerships, it is still the case that a majority of the efforts to reduce youth violence in communities are limited as to their intended audience or their approach. The intended targets of youth violence interventions are frequently violent offenders. The strategies used frequently involve identifying, incarcerating, and/or rehabilitating known juvenile offenders to prevent them from committing violent acts again. Although such criminal justice efforts are important, evidence-based primary prevention strategies have the potential to prevent youth violence from occurring in the first place. Many interventions are also limited by an approach that focuses solely on individual- or relationship-level factors. Research indicates that prevention activities should attend to the accumulation of risk factors across multiple levels of the social ecology, as youth with multiple risk factors are more likely to become violent than those exposed to only one risk factor (Herrenkohl et al., 2000). While it is important to pay attention to individual- and relationship-level factors (e.g., early aggressive behavior, social problem-solving skill deficits, negative parental influences, exposure to violence, and affiliation with delinquent peers), attention to the roles that larger sociocultural, economic, and community factors play in the development of youth violence is also important, particularly when attempting to generate a community-wide impact on reducing youth violence rates.

Unfortunately, most evidence-based prevention strategies tend to focus on addressing individual- and/or relationship-level risk factors for youth violence. A multifaceted prevention approach is needed to reduce risk factors and to enhance protective factors at the individual, relationship, and community levels. A multifaceted prevention approach includes complementary components (e.g., programs, policies, and strategies) that are designed to work at multiple levels of the social ecology to address identified needs within a community. Moreover, a prevention approach designed to have a community-wide impact on youth violence needs to provide an adequate exposure to the prevention components to a sufficiently large number of people to have the level of saturation necessary to achieve desired preventive effects. The type and intensity of the components provided are likely to vary according to the risk factors present in the groups involved in the program. By including universal components (i.e., delivered to all youth, regardless of risk) as well as components that focus on selective subgroups of youth or families at elevated risk, a multifaceted approach can have a pervasive reach within a community, thereby increasing the likelihood of community-wide reductions in youth violence. However, few studies have utilized rigorous methodologies to evaluate comprehensive and multifaceted efforts to prevent youth violence in specific communities.

## History of the National Centers of Excellence in Youth Violence Prevention

The National Centers of Excellence in Youth Violence Prevention (YVPCs) are filling some of these critical gaps by partnering with communities to prevent violence. The YVPCs were established in 2000 through a Congressional mandate in response to the 1999 Columbine school shootings, and they represent DVP’s largest investment in youth violence prevention (www.cdc.gov/violenceprevention/ACE; see Table 1 for a list of all current and former YVPCs). In 2000–2005, CDC funded ten centers (then known as the Academic Centers of Excellence [ACE] Program; David-Ferdon & Hammond 2008) across the U.S., with the primary goals of: (1) building the scientific infrastructure necessary to support the development and widespread application of effective youth violence interventions; (2) promoting interdisciplinary research strategies to address the problem of youth violence; (3) fostering collaboration between academic researchers and communities; and (4) empowering communities to address the problem of youth violence (CDC, 2004).

**Table 1:** 2000–2015 National centers of excellence in youth violence prevention program listing

2000–2005: Comprehensive Centers:	2000–2005: Developing Centers:	2005–2010: Academic Centers of Excellence in Youth Violence Prevention:	2005–2010: Urban Partnership: Academic Centers of Excellence:	2010–2015: National Centers of Excellence in Youth Violence Prevention:
Selected for their already established expertise in the field of youth violence prevention as well as their strong community linkages.	Focused on developing and implementing community response plans, training health care professionals and conducting small, pilot projects to evaluate effective interventions.	Focused on fostering relationships with local community partners to help develop, implement, and evaluate promising prevention efforts.	Selected in urban locations at high risk for youth homicide.	Implementing and evaluating a comprehensive youth violence prevention strategy.
(1) Columbia University	(1) University of California—Riverside	(1) Columbia University	(1) Children’s Hospital of Philadelphia	(1) Johns Hopkins University
(2) Harvard University	(2) University of California—San Diego	(2) Harvard University	(2) Meharry Medical College	(2) University of Chicago
(3) Johns Hopkins University	(3) University of Michigan	(3) Johns Hopkins University		(3) University of Colorado
(4) University of Alabama at Birmingham	(4) University of Puerto Rico	(4) University of California—Berkeley		(4) University of Michigan
(5) University of Hawaii at Manoa	(5) Virginia Commonwealth University	(5) University of California—Riverside		(5) University of North Carolina—Chapel Hill
		(6) University of Chicago		(6) Virginia Commonwealth University
		(7) University of Hawaii		
		(8) Virginia Commonwealth University		

During the second funding cycle (2005–2010), we asked YVPCs to partner with one defined community to achieve the objectives listed above and to monitor the magnitude and distribution of youth violence. Ten YVPCs were funded in the 2005–2010 cycle (CDC, 2004). Over the course of this first decade of funding, the objectives of the YVPC Program progressed to an increased emphasis on measuring and evaluating the public health impact of youth violence prevention efforts. In the 2005–2010 cycle, research emphasis was placed on efficacy and effectiveness research as well as dissemination/implementation research within local defined communities.

In the current, third cycle of funding (2010–2015), there are six YVPCs (see Table 2 for a list of these YVPCs and the characteristics of their intervention communities) that are implementing a multifaceted and comprehensive youth violence prevention approach. The theme of partnering with local, well-defined communities continues in the current round of YVPCs with an emphasis on incorporating state and local health departments as an important youth violence prevention partner. The goal of each Center is to reduce youth violence in one defined, high–risk community through the implementation and evaluation of a multifaceted, comprehensive, and evidence-based primary prevention approach. This comprehensive strategy was expected to include components with the following key characteristics:

**Table 2:** 2010–2015 Youth violence prevention centers community characteristics and interventions

Youth Violence Prevention Center	Intervention community	Intervention community characteristics	Comprehensive intervention components
Johns Hopkins University http://www.jhsph.edu/PreventYouthViolence	Lower Park Heights (Baltimore, MD)	Population size: 12,119 Prop of youth 10–24: 22 %	Safe Streets Problem Alcohol Outlet Monitoring Olweus Bullying Prevention Program Coping Power
University of Chicago http://www.ssa.uchicago.edu/ccyvp	Humboldt Park (Chicago, IL)	Percent at or below poverty line: 33 % Prop Black: 60 %	CeaseFire CeaseFire—School GREAT Schools and Families SAFE Children
University of Colorado http://www.colorado.edu/cspv/ace/	Montbello (Denver, CO)	Population size: 30,348 Prop Black: 28.4 % Prop Hispanic: 58.8 %	Promoting Alternative Thinking Strategies (PATHS) Strengthening Families Positive Family Support
University of Michigan http://yvpc.sph.umich.edu/	Durant-Tuuri-Mott (Flint, MI)	Population size: 5000 Prop Black: 80 % Percent at or below poverty line: 40 %	Youth Empowerment Solutions Fathers and Sons Clean and Green/Adopt-a-lot ED Brief Intervention Community Outreach Program (Boys and Girls Club) Community Mobilization
University of North Carolina https://ncace.web.unc.edu/	Lumberton County (Rural NC)	Population size: 129,000 Prop Native American: 38 % Percent at or below poverty line: 35 %	Positive Action (middle schools) Parenting Wisely Teen Court
Virginia Commonwealth University http://www.clarkhill.vcu.edu	Elkhardt, Boushall, and Thompson (Richmond, VA)	Prop Black: 60–94% Percent at or below poverty line: 62–85%	Olweus Bullying Prevention Program Staying Connected with Your Teen Parenting Wisely

1. Components directed at both universal and high-risk populations within the defined community.
2. Components directed at risk factors from each of the following levels of influence: individual (e.g., delinquency, substance abuse, lack of social skills); relationship (e.g., inadequate parental monitoring, supervision, discipline; peer norms supporting violence); and community (e.g., social disorganization, lack of cohesion, lack of economic or supervised recreational activities for youth). The prevention strategy components were intended to be complementary and to have sufficient reach and dosage to demonstrate a community-wide effect.
3. Components that have documented evidence of effectiveness. In other words, the program components must have demonstrated positive effects as described in a peer-reviewed journal article in which the program was evaluated using rigorous randomized or quasi-experimental designs.

**Fig. 1:**
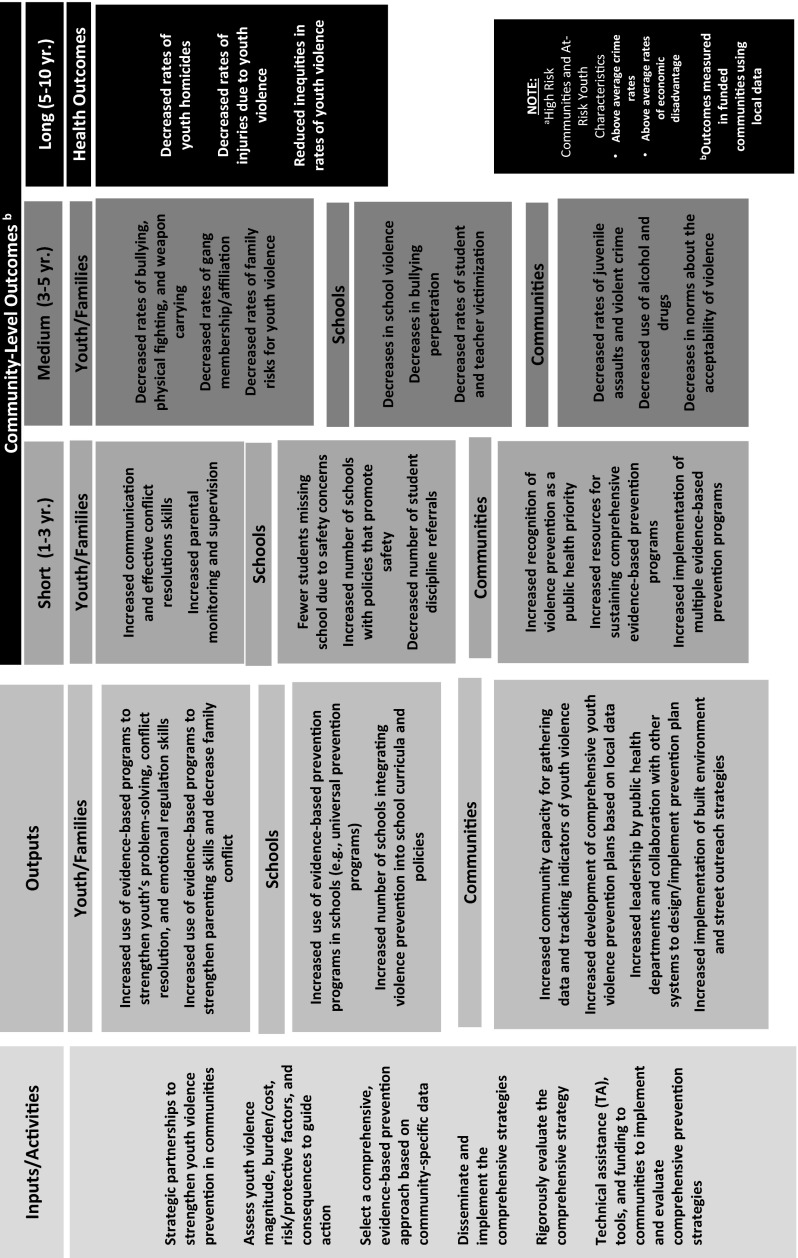
Conceptual framework for the CDC Centers of Excellence in Youth Violence Prevention (YVPCs) targeting high risk communities and at-risk youth^a^

Table 2 presents basic descriptive information on the current YVPCs, their intervention communities, and their comprehensive prevention approach. All Centers are also conducting a rigorous evaluation of their comprehensive approach in order to assess the impact of their prevention strategy on community-wide rates of violence. The rigorous evaluation approaches include interrupted time series and multiple baseline, or stepped wedge trial, designs (Farrell, Henry, Bradshaw, & Reischl, 2016). Below, we briefly describe the conceptual framework for the Centers, as depicted in Fig. 1. This framework is intended to be a visual depiction of the general key concepts and guiding principles that undergird the current work of the YVPCs, noting that there is considerable between-Center variability around the specific inputs/activities, outputs, and outcomes/impact that will be described in some detail within the articles contained within this special issue.

## Conceptual Framework

### Inputs/Activities

The first part of the conceptual framework describes the inputs necessary for effective youth violence prevention, and specifies the structural and relational conditions that should be in place to address youth violence within a specific community. They include: (a) forming strategic partnerships to strengthen youth violence prevention in communities; (b) assessing the magnitude, burden/cost, risk/protective factors, and consequences to guide action; (c) selecting a set of comprehensive, evidence-based prevention strategies based on community-specific data; (d) disseminating and implementing the comprehensive youth violence prevention strategies; (e) rigorously evaluating them; and, (f) providing technical assistance and funding to communities to develop, implement, and evaluate them in order to sustain youth violence prevention activities. These inputs highlight the importance of having strong university-community partnerships in prevention activities and basing community action on data and evidence-based practices. Furthermore, YVPC funding provides some of the infrastructure necessary to provide technical assistance to communities to become full partners in youth violence prevention, including technical assistance to support the rigorous evaluation of their prevention efforts. This special issue provides an in-depth description of all of these inputs and activities among the current YVPC grantees. We describe each of the articles in this special issue that mirror the inputs/activities of the YVPC Conceptual Framework below.

#### Assess Youth Violence Magnitude, Burden/Cost, Risk/Protective Factors, and Consequences to Guide Action

First, Masho and her colleagues describe each of the Centers’ youth violence surveillance efforts, including their primary (e.g., community surveys) and secondary (e.g., police and hospital data) data sources as well as their contextual data. They also discuss the ways in which the Centers analyze the data and disseminate them to communities in order to inform action. Masho, Schoeny, Webster, and Sigel (2016) suggest that many sources of surveillance data are relatively inexpensive and provide valuable information to guide intervention selection, and monitor and evaluate selected programs, policies, and strategies.

### Select a Comprehensive, Evidence-Based Prevention Approach Based on Community-Specific Data

Second, Kingston and her colleagues describe the methods that the Centers employed in order to select and assemble comprehensive packages of evidence-based strategies to prevent youth violence. A comprehensive approach uses multiple strategies that target change in community-specific risk and protective factors across multiple levels of the social ecology (i.e., individual, peer, family, school, and community). The investigators highlight the persistent gap between our youth violence prevention evidence base and the programs that are actually implemented in communities nationwide. Kingston, Bacallao, Smokowski, Sullivan, and Sutherland (2016) show how the YVPCs have collaborated with their community partners in choosing and assembling a comprehensive evidence-based youth violence prevention strategy.

#### Rigorously Evaluate the Comprehensive Strategy

Third, Farrell and his colleagues discuss the designs of strategies that the Centers have devised in order to rigorously evaluate the impact of their approaches to youth violence. In this paper, the authors describe the challenges in conducting rigorous evaluations of comprehensive, community-level interventions and the approach that each of the YVPCs has taken to develop a rigorous design to ensure that any changes in youth-violence-related community-level outcomes can be attributed to the activities of the Centers.

#### Strategic Partnerships and Technical Assistance to Strengthen Youth Violence Prevention in Communities

Finally, Morrel-Samuels and her colleagues describe the process of community engagement in youth violence prevention activities. The authors discuss the methods for community engagement across three of the YVPCs representing urban, suburban, and rural communities, and provide details concerning community-level barriers and facilitating factors that influence the formation and implementation of comprehensive, evidence-based youth violence prevention strategies. Morrel-Samuels, Bacallao, Brown, Bower, and Zimmerman (2016) also provide some recommendations, based on the experience of the Centers, on ways to successfully integrate community engagement in violence prevention initiatives.

### Outputs

Through the specified inputs and activities, the outputs (as illustrated in the conceptual framework) lay out the intended results of the Center activities that are expected to lead to ultimate short- and long-term reductions in youth violence. The conceptual model includes outputs involving youth/families, schools, and communities. At the youth/family, school, and community levels, important outputs include the increased use of evidence-based programs to address youth, parental, school, and community needs. For example, evidence suggests that parenting/family programs that strengthen caregivers’ use of consistent discipline and decrease family conflict are effective at reducing youth violence (Kaminski, Valle, Filene, & Boyle, 2008). Given the activities of the YVPCs, it is expected that there will be an increased use of this evidence targeted at families in high-risk communities.

Furthermore, because the YVPCs partner with communities to address youth violence, additional community-level outputs include increases in: (a) community capacity for gathering data and tracking indicators of youth violence; (b) the development of comprehensive youth violence prevention plans based on local data; and (c) leadership by public health departments and collaboration with other systems to design/implement the prevention plan. All of these outputs are important factors in sustaining violence prevention activities and enhancing community capacity to utilize data to understand and respond to the needs of their community members.

### Outcomes/Impact

All of these inputs/activities and outputs come together to affect short-, medium-, and long-term community-level outcomes. The short-term outcomes include a host of behaviors that the comprehensive prevention strategy is designed to change. In the parent/family program discussed above, evidence-based family programs are designed to strengthen parenting skills. In turn, short-term outcomes include increased parental monitoring and supervision. Medium-term outcomes include the youth violence-related outcomes specifically targeted by the evidence-based programs. In the family example, this includes decreased rates of family risks for youth violence (e.g., family violence). Finally, long-term outcomes include the serious forms of youth violence-related outcomes that represent the biggest morbidity and mortality burdens to youth between the ages of 10 and 24 (CDC 2010)—youth homicide, violence-related injuries, and inequities in rates of youth violence. The current YVPCs are tracking these youth violence-related long-term outcomes and will rigorously evaluate whether their activities reduce these serious forms of youth violence.

## Conclusion

Many factors influence the prevalence of youth violence, and a comprehensive, evidence-based approach is required in order to address these factors. Research institutions, community organizations, and community members all play a critical role in understanding and addressing the level of violence in a community. The YVPCs and their community partners are collaborating to reduce youth violence by identifying, implementing, and evaluating comprehensive evidence-based prevention programs that utilize data to understand and tailor their prevention approaches. The YVPCs’ rigorous evaluation findings can also, in turn, contribute to the evidence base, thus advancing the field of youth violence prevention. YVPC activities and community partnerships are intended to promote the community-wide reach and dosage of evidence-based programs, establish an enhanced capacity for prevention, and improve understanding of rigorous community-level evaluation methods that will inform whether a community’s comprehensive, evidence-based approach is having an impact on community-wide rates of youth violence.
